# Gastroprotective Effect of Sinapic Acid on Ethanol-Induced Gastric Ulcers in Rats: Involvement of Nrf2/HO-1 and NF-κB Signaling and Antiapoptotic Role

**DOI:** 10.3389/fphar.2021.622815

**Published:** 2021-02-25

**Authors:** Mohammad Raish, Mudassar Shahid, Yousef A. Bin Jardan, Mushtaq Ahmad Ansari, Khalid M. Alkharfy, Abdul Ahad, Ibrahim Abdelsalam Abdelrahman, Ajaz Ahmad, Fahad I. Al-Jenoobi

**Affiliations:** ^1^ Department of Pharmaceutics, College of Pharmacy, King Saud University, Riyadh, Saudi Arabia; ^2^ Department of Pharmacology and Toxicology, College of Pharmacy, King Saud University, Riyadh, Saudi Arabia; ^3^ Department of Clinical Pharmacy, College of Pharmacy, King Saud University, Riyadh, Saudi Arabia

**Keywords:** sinapic acid, ethanol, oxidat ive stress, inflammation, Nrf2/HO-1 signaling pathway, gastric mucosal lesions

## Abstract

**Background: **In the current study, we evaluated the therapeutic potential of sinapic acid (SA) in terms of the mechanism underlying its gastroprotective action against ethanol-induced gastric ulcers in rats.

**Methods: **These effects were examined through gross macroscopic evaluation of the stomach cavity [gastric ulcer index (GUI)], alteration in pH, gastric juice volume, free acidity, total acidity, total gastric wall mucus, and changes in PGE2. In addition, we evaluated lipid peroxidation (malondialdehyde), antioxidant systems (catalase and glutathione), inflammatory markers [tumor necrosis factor-α (TNF-α) and interleukin-6 (IL-6), and myeloperoxidase (MPO)], apoptotic markers (caspase-3, Bax, and Bcl-2), nuclear factor-κB [NF-κB (p65)], NO levels, and histopathological staining (H and E and PAS).

**Results: **In rats with ethanol-induced ulcers, pre-treatment with SA (40 mg/kg p. o.) decreased the sternness of ethanol-induced gastric mucosal injuries by decreasing the GUI, gastric juice volume, free acidity, and total acidity. In addition, the pH and total gastric mucosa were increased, together with histopathological alteration, neutrophil incursion, and increases in PGE2 and NO_2_. These effects were similar to those observed for omeprazole, a standard anti-ulcer drug. SA was shown to suppress gastric inflammation through decreasing TNF-α, IL-6, and MPO, as well as curbing gastric oxidative stress through the inhibition of lipid peroxidation (MDA) and restoration of depleted glutathione and catalase activity. SA inhibited Bcl-2-associated X (Bax) and caspase-3 activity, and restored the antiapoptotic protein Bcl-2; these findings indicate the antiapoptotic potential of SA, leading to enhanced cell survival. SA also repressed NF-κB signaling and increased IκBα. Moreover, SA upregulated the nuclear factor erythroid 2-related factor 2 (Nrf2) and heme oxygenase-1 (HO-1), thereby restoring depleted antioxidant defense enzymes and implicating the NRF2/HO-1 signaling pathways.

**Conclusion: **These results suggest that the prophylactic administration of SA (40 mg/kg) can ameliorate ethanol-induced gastric ulcers in rats primarily via the modulation of Nrf2/HO-1 and NF-κB signaling and subsequent enhancement of cell viability.

**GRAPHICAL ABSTRACT:**
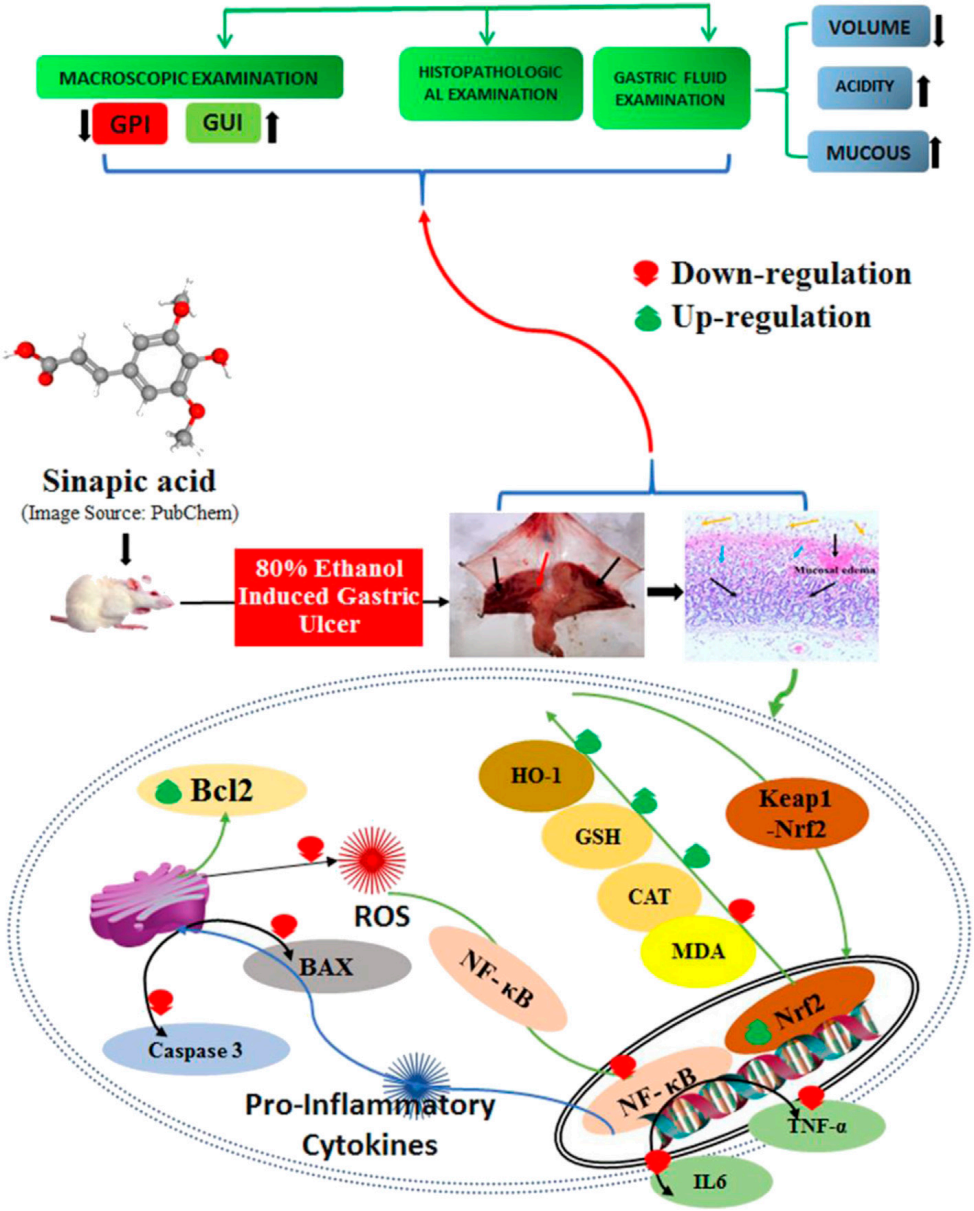


## Introduction

Gastric ulcers (GUs) and duodenal ulcers (DUs) are common human gastric intestinal illnesses with a high morbidity of approximately 5–10% over a lifetime, and thus represent leading health issues (Lanas and Chan, 2017). The origin and cause of GUs and DUs remains controversial; however, they are considered to arise from an imbalance between mucosal invasive factors and the protective factors of gastric mucosa, which together result in the destruction of the mucosal protective barrier, leading to ulcer formation (Franke et al., 2005; Zhou et al., 2020). The mucosal lining is often exposed to invasive factors, such as NSAIDs, antibiotics, smoking, irritant chemicals, and alcohol; these factors can cause GUs because of the destruction of the protective mucosal barrier as a result of bicarbonate secretion, mucus, prostaglandins, cell regeneration, and endogenous antioxidants (Ibrahim et al., 2016; Borgquist et al., 2018). Hence, the therapeutic scheme for gastroprotective anti-ulcer treatment involves either intensifying protective barriers and/or reducing gastric mucosal invasive factors. Ulcerative gastric lesions are often linked to the use of drugs, including NSAIDS, antibiotics, antipsychotics, antidepressants, and alcohol (Philpott et al., 2014).

The ethanol-induced gastric ulcer rodent model is widely used to study acute gastritis (Cho and Ogle, 1992). Many studies have demonstrated that the physiological and morphological alterations in the gastric acid, mucus, and hemorrhagic and necrotic foci of the experimental animals are similar to those observed in humans (Fu et al., 2018). The accumulation of ROS/RNS induces oxidative stress and inflammation in the gastric mucosa, and has been implicated in the formation of gastric lesions (Oates and Hakkinen, 1988; Wu et al., 2018; Aziz et al., 2019). The accumulation of ROS/RNS oxidizes lipids and proteins; therefore, mucosal barriers enhance the gut permeability, stimulate macrophages, and increase inflammatory cytokine release (TNFα and IL-6) and NF-kB signaling, leading to ulcerative gastritis. Ulcerative gastritis is characterized by inflammation and mucosal ulceration, bleeding, and perforation (Oates and Hakkinen, 1988). The nuclear factor erythroid 2-related factor 2 (Nrf2) and hemoxygenase-1 (HO-1) play critical roles in gastrointestinal protection by restoring the antioxidant defense (Silva-Islas and Maldonado, 2018; Yanaka, 2018). In addition, it has been demonstrated that Nrf2 also suppresses NF-κB, consequently reducing proinflammatory cytokine signaling and activating NRF2 and HO-1. Therefore, these molecules and pathways exhibited a protective role against gastric ulceration induced by ethanol and other insults. Furthermore, ROS/RNS increase oxidative-stress-induced apoptosis in gastric mucosal cells (Tayeby et al., 2017). Moreover, ethanol promotes hypersecretion of gastric acid, proinflammatory cytokines, and ROS/RNS, which together function to induce apoptosis and suppress the production of NO and prostaglandin E2 (Laloo et al., 2013; Antonisamy et al., 2014; Albaayit et al., 2016). The gaseous chemokine NO, which is a vasodilator that is synthesized from arginine by two molecules of iNOS, functions to stimulate blood capillaries to increase blood and has anti-inflammatory and gastric-healing effects. iNOS induction may promote GUs (Cho, 2001).

Natural herbs and their phytoconstituents with potent antioxidant, anti-inflammatory, and antiapoptotic effects may offer good gastrointestinal protection. Sinapic acid (SA) has potent antioxidant, anti-inflammatory, and antiapoptotic activities (Raish et al., 2018a; Bin Jardan et al., 2020). The current article reports the first *in vivo* study of the gastroprotective effects of SA and the underlying mechanism of gastric protection in ethanol-induced GUs in rats.

## Materials and Methods

### Chemicals

Sodium azide was obtained from Sigma Chemicals Co. (St. Louis, MO, United States). Tumor necrosis factor-α (TNF-α), interleukin-1β (IL-1β), prostaglandin E2 (PGE2), nitric oxide (NO), MDA, GSH, and CAT activity kits were procured from Sigma Chemicals Co. (St. Louis, MO, United States). Antibodies against caspase-3, Bcl-2-associated X (Bax), Bcl-2, NF-κB (p65), IκB-α (p65), NRF2, HO-1, TGF β, and β-actin, as well as a horseradish peroxidase-conjugated secondary antibody, were purchased from Santa Cruz Biotechnology (Santa Cruz, CA, United States). The NE-PER Nuclear and Cytoplasmic Extraction Kit was obtained from Pierce Biotechnology (Rockford, IL, United States). The enzyme-linked immunosorbent assay (ELISA) kits for rat TNF-α, IL-6, and MPO were obtained from R&D Systems (Minneapolis, MN, United States). All other chemicals were analytical grade and were obtained from standard commercial suppliers.

### Animals

Male Wistar rats (170–202 g) were acquired from the Central Animal House Facility of King Saud University and were kept in animal cages with a 12-h light and dark cycle at 25 ± 2°C. The rats were fed standard rat chow and provided water ad libitum. The study was approved by the Research Ethics Committee of College of Pharmacy, King Saud University (KSU-SE-20–43). The safe effective dose was estimated, and an acute oral toxicity study was carried out as per the OECD guidelines using the fixed dose method (OECD, 2000), as described previously [8]. Three clinical doses were selected based on previous literature, and the dose response was examined for anti-gastric ulcer activity in rats with ethanol-induced gastritis that were orally treated with 10, 20, and 40 mg/kg SA for 7 days. The optimal anti-gastric ulcer dose was determined by examining the gastric ulcer index (GUI) and PGE2 content.

### Acute Toxicity

The acute oral toxicity was monitored using the limit test procedure as per OECD test guidelines on acute oral toxicity test 401 (Walum, 1998). A total of 12 overnight fasted rats of either sex were used in this investigation. The rats were divided into two groups (n = 6 each): the vehicle group, consisting in 1% CMS in normal saline, and the SA group, consisting in 2,000 mg/kg SA in 1% CMC normal saline solution, administered by oral gastric gavage. The rats were not fed for 3 h following the administration of the solutions. The rats were observed uninterruptedly for 30 min and then every half hour for 4 h for any major behavioral changes and general motor signs, as well as up to 72 h for any mortality. Significant behavioral changes and mortality were observed (data not shown).

### Experimental Design

The Wistar male rats were arbitrarily assigned into four groups (GP1–4; n = 6 each). Rats in GP1, GP2, and GP4 were administered normal saline orally for 7 days using gastric gavage, whereas those in GP3 were treated orally with SA (40 mg/kg body weight) in normal saline for 7 days (Almasaudi et al., 2016). Gastric ulceration was induced on the last day by intubating 80% ethanol (5 ml/kg b. w.) into rats in GP 2–4. The rats in GP3 and GP4 were administered SA (40 mg/kg body weight) in normal saline and omeprazole (20 mg/kg b. w) in normal saline, a proton pump inhibitor orally 2 h before ethanol administration on day 7 (Huang et al., 2013). The animals were euthanized under anesthesia (ketamine 80–100 mg/kg IP and xylazine 10–12.5 mg/kg IP in 0.9% saline) 3 h after ethanol administration, and their stomachs were collected.

### Gastric Ulcer Index

The stomach sacs were opened along the greater curvature, washed and rinsed with cold normal saline, blotted dry between filter paper sheets, and pinned flat on cardboard for examination of gross lesions. The GUI was estimated in accordance with the method of Guth et al. (Guth et al., 1979). Each gastric cavity was scrutinized wholly, and the degree of ulceration was graded as follows (Kunchandy et al., 1985; Haule et al., 2012): 0, no lesions (normal stomach); 0.5, hyperemia (red coloration); 1, hemorrhagic spots; 2, 1–5 small ulcers; 3, many small ulcers; 4, many small and large ulcers; 6, stomach full of ulcers with perforations. The protective index (PI) was calculated using the following formula:

$$PI=\frac{ulcer\;model\;-\;ulcer\;treated}{ulcer\;model\;}\times 100\;\;$$

### Assessment of Gastric Juice Acidity

The stomach contents were collected into centrifuge tubes and successively centrifuged at 4,000 rpm for 10 min, and the supernatant was titrated for pH measurement with a 0.1 mM NaOH solution (Saremi et al., 2019).

### Assessment of Gastric Wall Mucus

Gastric wall mucus (GWM) was estimated as described by Corne et al. (Corne et al., 1974). Briefly, the glandular part of the stomach was detached and submerged in 10 ml of 0.1% w/v Alcian blue for 2 h. The excess dye was then removed using 10 ml of a 0.25 M of sucrose solution, and the remaining dye was washed out using 10 ml of 0.5 M MgCl_2_ for 30 min. Subsequently, 4 ml of diethyl ether was added to the extract, followed by incubation with shaking for 2 min and centrifugation at 4,000 rpm for 10 min. The absorbance of each group was estimated at 580 nm.

### Measurement of Oxidative Stress and Antioxidant Enzymes

The lipid peroxidation (MDA) level in gastric tissues was examined as per the method of Ohkawa et al., 1979 (Ohkawa et al., 1979) using a colorimetric assay kit (Sigma Aldrich). The level of reduced glutathione (GSH) and catalase in gastric tissues was assessed by the standard commercial kits according to the manufacturer’s protocols (sigma Aldrich) using the methods of Akerboom et al. (Akerboom and Sies, 1981) and Goth et al. (Goth, 1991).

### Inflammatory Markers and Cytokines

The levels of inflammatory markers (PGE2 and NO) and proinflammatory cytokines (TNF-α, IL-6, and MPO) in the gastric tissue homogenates were measured using ELISA kits obtained from R&D Systems. Measurement of marker absorbance was performed at a wavelength of 450 nm (Al-Yahya et al., 2016).

### Western Blotting

Western blotting was performed for NRF2, HO-1, caspase-3, Bax, Bcl2, TGF-β, NF-κB (p65), and IκBα as described previously (Raish et al., 2018a; Raish et al., 2018b). Gastric tissue cytosolic and nuclear proteins were extracted using an NE-PER™ Nuclear and Cytoplasmic Extraction Reagents kit (Thermo Fisher Scientific). The total protein levels in the cytoplasmic and nuclear fractions were estimated by the bicinchoninic acid method using a Pierce™ BCA Protein Assay Kit (Thermo Fisher Scientific) (Smith et al., 1985). Immunoblots were prepared as per the procedures of Towbin et al. (Towbin et al., 1979). Briefly, 30 μg of protein was electrophoresed on 10% SDS− polyacrylamide gels, transferred to activated PVDF membranes, and blocked in a blocking buffer (4% skim milk and BSA in TBS of 1% Tween 20). Subsequently, the membrane was incubated overnight (4°C) with antibodies against NRF2, HO-1, caspase-3, Bax, Bcl2, NF-κB (p65), IκBα, TGF-β, or β-actin. After repeated washing steps with 1% Tween TBS and TBS, the membrane was incubated with the appropriate secondary antibodies for 2 h (room temperature). The membranes were then washed again with TBST four times for 5 min each. Bands were scanned using Luminata™ Western Chemiluminescent horse radish peroxidase Substrates (Millipore, Billerica, MA, United States), followed by densitometric analysis of the immunoblots [LI-COR C-Di-Git Blot Scanners (Lincoln, NE, United States)].

### Histopathology

Gastric tissues were fixed in 10% formalin buffer, embedded in paraffin, cut into 5-μm sections, stained with hematoxylin and eosin (H and E) and Periodic acid Schiff (PAS) stain solution, and examined under a light microscope for histological and mucosal evaluations (Raish et al., 2018b).

### Statistical Analysis

All data are expressed as the mean ± SEM. Statistical analysis was performed using Graph Pad Prism 9 (Graph Pad Software Inc., CA, United States). Shapiro–Wilk test was performed to decide normality. One-way analysis of variance (ANOVA) with Dunnett’s test was to analyze the parametric data and Kruskal–Wallis comparisons test for non-parametric data. *p*-values < 0.05 were considered statistically significant.

## Results

### Acute Toxicity

No mortality or signs of toxic effects were observed as per the guideline of OECD when 2,000 mg/kg b. w. was selected for acute toxicity; therefore, SA seems to be nontoxic in rats (data not shown).

### Gross Visual Inspection of the Rat Gastric Mucosa

**FIGURE 1:**
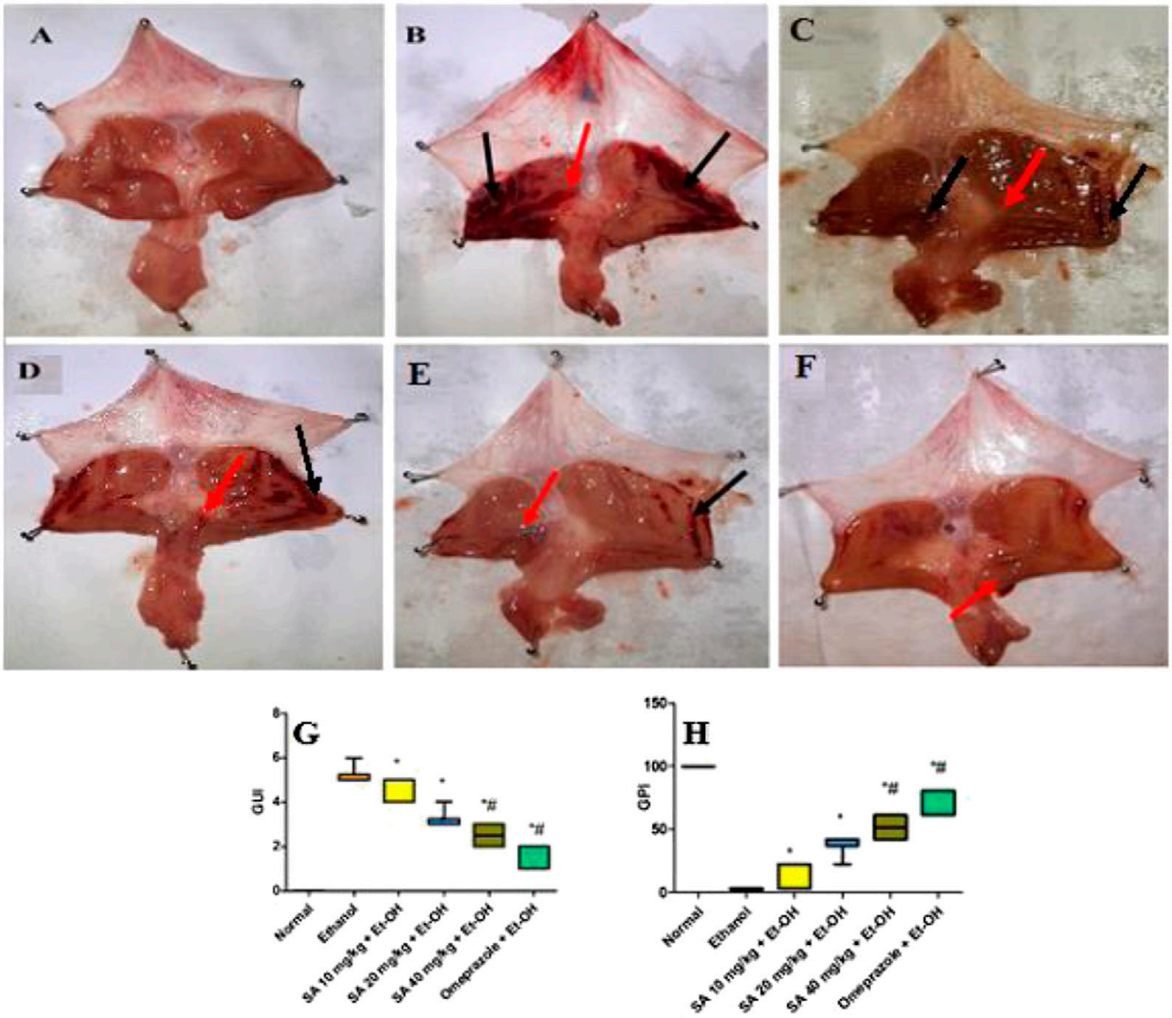
Gross visual inspection of the rat gastric mucosa **(A)** The normal control group exhibited a normal gastric mucosal tissue architecture **(B)** Ethanol-administered rats showed extensive and severe hemorrhagic gastric mucosal lesions with a GUI of five and a GPI of 0% **(C)** The SA (10 mg/kg) pre-treated ethanol-induced rats exhibited hemorrhagic lesions of the gastric mucosa compared with normal control animals, with a GUI of 4.67 ± 0.21 and a GPI of 9.66 ± 4.08% **(D)** The SA (20 mg/kg) pre-treated ethanol-induced rats exhibited hemorrhagic lesions of the gastric mucosa compared with normal control animals, with a GUI of 3.17 ± 0.17 and a GPI of 38.70 ± 3.23% **(E)** The SA (40 mg/kg) pre-treated ethanol-induced rats exhibited mild hemorrhagic lesions of the gastric mucosa compared with normal control animals, with a GUI of 2.55 ± 0.22 and a GPI of 50.32 ± 4.32% **(F)** The omeprazole (20 mg/kg) pre-treated ethanol-induced rats exhibited no hemorrhagic lesions, with slight inflammation of the gastric mucosa compared with normal control animals, with a GUI of 1.33 ± 0.21 and a GPI of 72.90 ± 4.08% black arrow = hemorrhagic lesions; red arrow = inflammation **(G)** Box plot of gastric ulcer index (GUI) and **(H)** gastric percentage index of ethanol-induced ulcers (GPI). Data for of SA 10 and 20 mg/kg bw ethanol treated group obtained from pilot study. Non-parametric data of box plots are presented and Kruskal-Wallis analysis of variance is performed to compare groups. *Denotes significant differences compared to the ethanol-induced gastric ulcer group (*p* < 0.05); #denotes significant differences compared to the normal control.

The ethanol (Et-OH)-induced gastric ulcer GP demonstrated severe ulcer injuries and widespread visible hemorrhagic lesions of the gastric mucosa (Figure 1). In contrast, the rats that received saline showed no signs of hemorrhagic lesions or ulceration compared with the gastric ulcer group (Et-OH group). Compared with the normal control group (normal control), the GUI of the ethanol-treated group was increased to 5. Moreover, compared with the Et-OH-treated group, pre-treatment with omeprazole (20 mg/kg) and SA (40 mg/kg) significantly (*p* < 0.05) reduced the GUI to 1.33 and 2.5, respectively (Table 1). However, the GUI of the omeprazole (20 mg/kg) and SA (40 mg/kg) groups was 72.90 ± 4.08% and 50.32 ± 4.32% (*p* < 0.05), respectively, compared with the Et-OH-treated group. These results demonstrate that omeprazole and SA offer significant protection against ethanol-induced gastric ulceration. The GUI and gastric percentage index of ethanol-induced ulcers (GPI) were analyzed as nonparametric data represented as box plots, and a Kruskal−Wallis analysis of variance was performed to compare the groups. In the graphs, “*” denotes significant differences compared with the ethanol-induced gastric ulcer group (*p* < 0.05); #denotes significant differences compared with the normal control.

**TABLE 1:** Gastric Ulcer Index, Gastric Protective Index, and PGE2 of SA (10, 20, and 40 mg/kg) and omeprazole (20 mg/kg) pre-treated rats.

Groups	Gastric ulcer Index ± SEM	Gastric protective Index ± SEM	Gastric mucosal PGE2 (Pg/mg)
Normal	0.00 ± 0.00	99.98 ± 0.00	348.77 ± 3.82
Ethanol	5.166 ± 0.00	−0.01 ± 3.23	151.72 ± 4.84
SA 10 mg/kg + Et-OH	4.67 ± 0.21	9.66 ± 4.08	198.56 ± 3.78
SA 20 mg/kg + Et-OH	3.17 ± 0.17	38.70 ± 3.23	216.00 ± 3.33
SA 40 mg/kg + Et-OH	2.50 ± 0.22*#	51.60 ± 4.33*#	239.33 ± 2.20
Omeprazole 20 mg/kg + Et-OH	1.33 ± 0.21*#	74.18 ± 4.08*#	272.33 ± 4.47

Non-parametric data of box plots are presented and Kruskal-Wallis analysis of variance is performed to compare groups. *Denotes significant differences compared to the ethanol-induced gastric ulcer group (*p* < 0.05); #denotes significant differences compared to the normal control.

The nonparametric data of box plots are presented and a Kruskal−Wallis analysis of variance was performed to compare the groups. *Denotes significant differences compared with the ethanol-induced gastric ulcer group (*p* < 0.05); #Denotes significant differences compared with the normal control.

### Effect of SA on Gastric Wall Mucus and Gastric Juice Acidity

A significant increase in volume, free acidity, and total acidity, and a reduction in the pH of the gastric juice were observed in rats with ethanol-induced ulcers compared with normal rats (107.77, 30.96, 24, and 53%, respectively). However, SA and omeprazole pre-treatment in ethanol-induced ulcerated rats significantly decreased the volume (48.71%, *p* < 0.05 and 77.02%, *p* < 0.05, respectively), free acidity (45.85%, *p* < 0.05 and 56.88%, *p* < 0.05, respectively), and total acidity (35.48%, *p* < 0.05 and 42.20, *p* < 0.05, respectively), and enhanced the pH of gastric juice compared with ethanol-induced ulcerated rats without pre-treatment (Table 2). The GWM was significantly decreased to 52.61% (*p* < 0.05) in the ethanol-induced ulcerated rats compared with the normal control rats. However, pre-treatment with SA and omeprazole significantly increased this parameter to 55.39% (*p* < 0.05) and 77.03% (*p* < 0.05), respectively, compared with ethanol-induced ulcerated rats without pre-treatment.

**TABLE 2:** Effect of SA on the pH, gastric juice volume, free gastric acidity, total gastric acidity, and gastric wall mucus content.

Groups	pH	Gastric juice volume	Gastric juice free acidity (meq/L)	Gastric juice total acidity (meq/L)	Gastric wall mucus (µg/gm)
Normal	2.42 ± 0.060	1.50 ± 0.10	253.84 ± 16.35	300 ± 19.32	373.53 ± 4.91
Ethanol	1.28 ± 0.083	3.12 ± 0.11	332.44 ± 11.49	372 ± 12.39	177.01 ± 5.61
SA 40 mg/kg + Et-OH	2.08 ± 0.108^*#^	1.60 ± 0.08^*#^	180.00 ± 8.71^*#^	240 ± 11.61^*#^	275.06 ± 7.39^*#^
Omeprazole 20 mg/kg + Et-OH	2.47 ± 0.088^*#^	0.72 ± 0.06^*#^	143.33 ± 12.02^*#^	215 ± 18.02*#	302.74 ± 3.31^*#^

Data are presented as the mean ± SEM (n = 6) per group. *Denotes significant differences compared with the ethanol-induced gastric ulcer group (*p* < 0.05); #denotes significant differences compared with the normal control group.

### Effect of SA on Prostaglandin E2 and Nitric Oxide Levels

**FIGURE 2:**
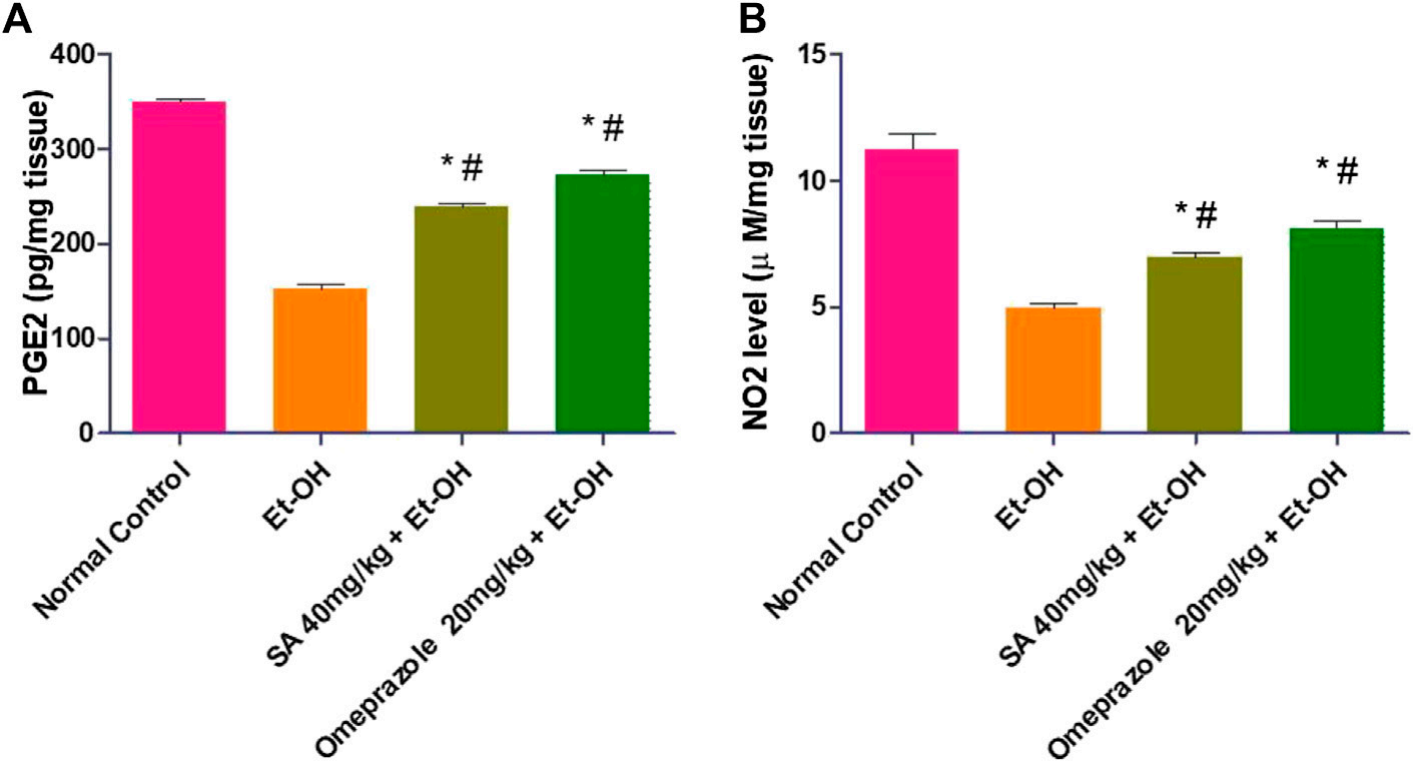
Effect of SA pre-treatment on **(A)** PGE2 and **(B)** NO2 levels in ‐treated rats. Data are presented as the mean ± SEM (n = 6) per group. ^*^Denotes significant differences compared with the ethanol‐induced gastric ulcer group (*p* < 0.05); #denotes significant differences compared with the normal control group.

Oral administration of ethanol significantly decreased the gastric mucosal PGE_2_ and NO levels by 56.49% (*p* < 0.05) and 55.78% (*p* < 0.05), respectively, compared with the normal control level. The gastric PGE_2_ and NO level was significantly increased after pre-treatment with SA and omeprazole (57.75%, *p* < 0.05 and 79.50% *p* < 0.05 for PGE_2_; 39.55%, *p* < 0.05 and 79.50%, *p* < 0.05 for NO, respectively) compared with ethanol-induced ulcerated rats without pre-treatment (Figure 2).

### Effect of SA Pre-treatment on Gastric Mucosal Inflammatory Parameters in Ethanol-Treated Rats (Figure 3)

**FIGURE 3:**
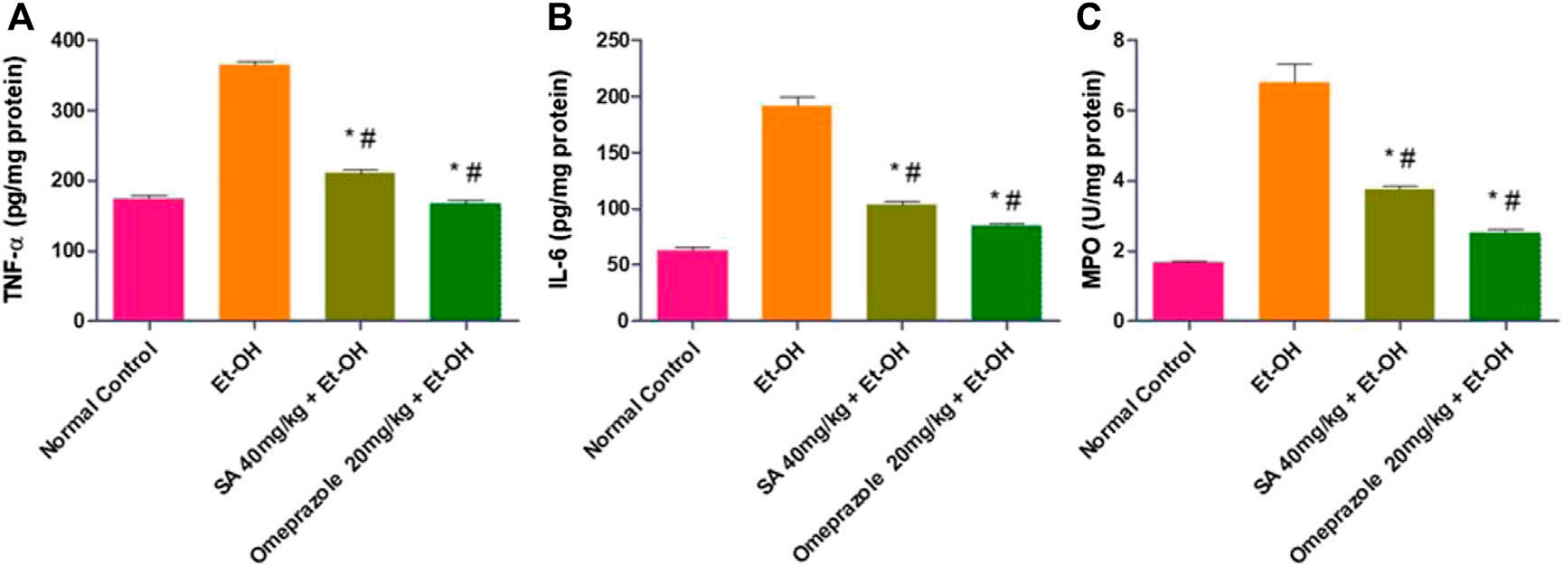
Effect of SA pre-treatment on gastric mucosal inflammatory parameters **(A)** TNF-α **(B)** IL-6 and **(C)** MPO in ethanol-treated rats. Data are presented as the mean ± SEM (n = 6) per group. ^*^Denotes significant differences compared with the ethanol-induced gastric ulcer group (*p* < 0.05); #denotes significant differences compared with the normal control group.

The levels of TNF-α and IL-6 in the gastric mucosa were significantly enhanced in ethanol-induced ulcerated rats compared with normal control rats (108.50%, *p* < 0.05 and 208.44%, *p* < 0.05, respectively). SA and omeprazole pre-treatment significantly reduced the levels of TNF-α and IL-6 by 42.11% (*p* < 0.05) and 54.06% (*p* < 0.05) for TNF-α and 46.16% (*p* < 0.05) and 55.63% (*p* < 0.05) for IL-6, respectively, compared with ethanol-induced rats without pre-treatment (Figure 3). The level of myeloperoxidase (MPO), a marker of gastric inflammation, was significantly increased by 303.97% (*p* < 0.05) compared with the normal control rats. Moreover, the increased neutrophil influx was substantially mitigated by pre-treatment with SA and omeprazole, by 44.71% (*p* < 0.05) and 63% (*p* < 0.05), respectively, for MPO (Figure 3).

### Effect of SA on Lipid Peroxidation and Antioxidant Enzymes

**FIGURE 4:**
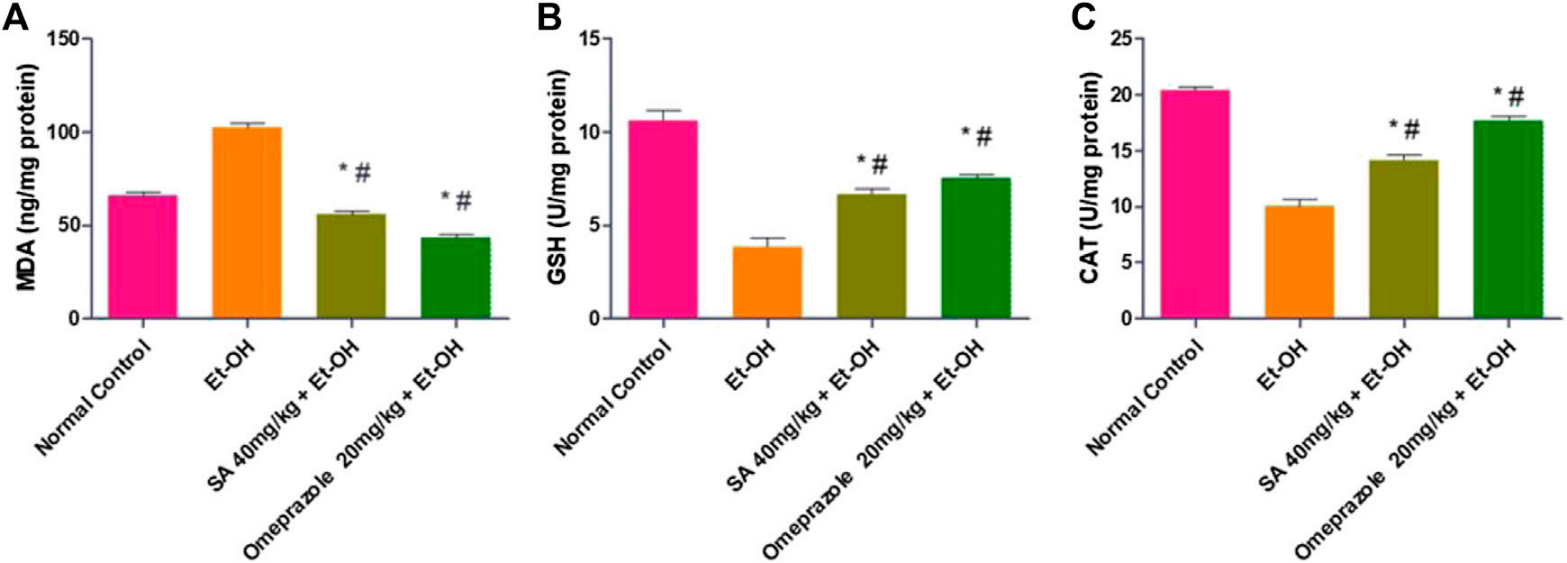
Effect of SA pre-treatment on lipid peroxidation and antioxidant enzymes **(A)** MDA **(B)** GSH and **(C)** Catalase in ethanol-treated rats. Data are presented as the mean ± SEM (n = 6) per group. ^*^Denotes significant differences compared with the ethanol-induced gastric ulcer group (*p* < 0.05); #denotes significant differences compared with the normal control group.

The lipid peroxidation level (MDA) was significantly increased by 141.47% (*p* < 0.05) in the gastric mucosa of ethanol-induced ulcerated rats compared with normal control rats. Pre-treatment with SA and omeprazole significantly reduced the enhanced level of MDA by 45.27% (*p* < 0.05) and 57.51% (*p* < 0.05), respectively, compared with ethanol-induced ulcerated rats without pre-treatment. The levels of defense antioxidant enzymes, such as GSH and catalase, were significantly depleted by 63.74% (*p* < 0.05) and 50.81% (*p* < 0.05), respectively, in ethanol-induced ulcerated rats compared with the normal control rats. These depleted levels were replenished by pre-treatment with SA and omeprazole, by approximately 73.34% (*p* < 0.05) and 95.11% (*p* < 0.05) for GSH and 41.00% (*p* < 0.05) and 76.36% (*p* < 0.05) for catalase, respectively compared with ethanol-induced ulcerated rats (Figure 4).

### Effect of SA Pre-treatment on Gastric Mucosal Apoptotic Markers in Ethanol-Treated Rats

**FIGURE 5:**
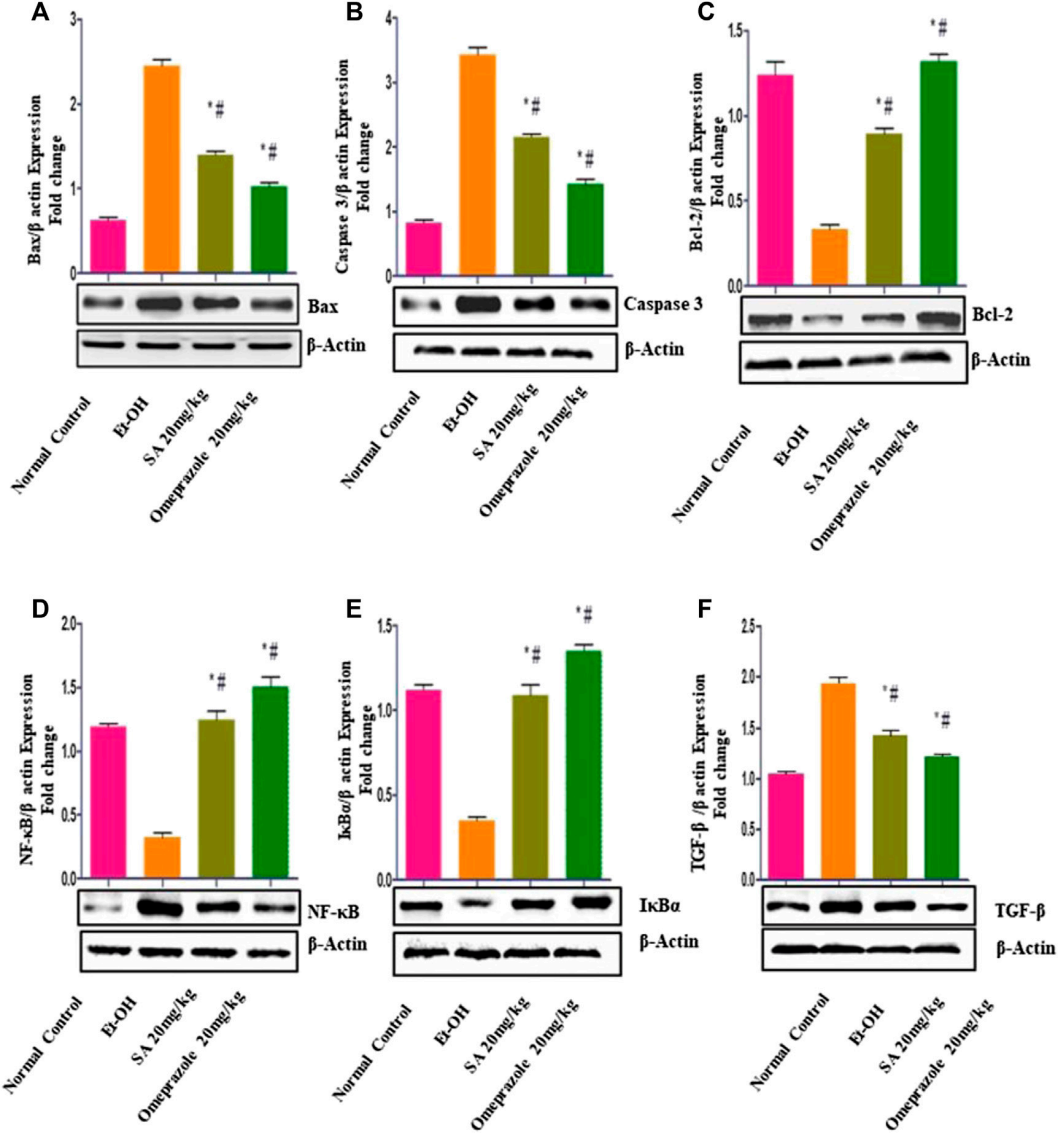
Effect of SA on the expression of **(A)** proapoptotic Bax **(B)** caspase-3 **(C)** antiapoptotic Bcl-2 **(D)** nuclear factor kappa [NF-κB (p65)] **(E)** nuclear factor of kappa light polypeptide gene enhancer in B-cells inhibitor (IκBα), and **(F)** transforming growth factor beta (TGF-β) in rats with ethanol-induced gastric ulcer rats. Data are presented as the mean ± SEM (n = 6) per group. *Denotes significant differences compared with the ethanol-induced gastric ulcer group (*p* < 0.05); #denotes significant differences compared with the normal control group.

The results illustrated in Figure 5 demonstrate that ethanol treatment induced apoptotic injuries in gastric mucosa, as demonstrated by the significant upregulation of the proapoptotic proteins Bax (*p* < 0.05) and caspase-3 (*p* < 0.05) and downregulation of the antiapoptotic protein Bcl2 (*p* < 0.05) compared with the normal control group. SA and omeprazole significantly reduced Bax (*p* < 0.05) and caspase-3 (28%) (*p* < 0.05) levels, and enhanced the expression of Bcl2 () (*p* < 0.05) relative to the ulcerated content (*p* < 0.001). Ethanol intubation significantly enhanced the nuclear translocation of the p65 subunit of NF-κB and downregulated IκBα relative to normal control rats. SA 40 mg/kg and omeprazole 20 mg/kg pre-treatment significantly reduced the expression of NF-κB (p65) and increased that of IκBα compared with ulcerated rats (Figure 5).

### Effect of SA Pre-treatment on Gastric Mucosal NRF2 and HO-1 Levels in Ethanol-Treated Rats

**FIGURE 6:**
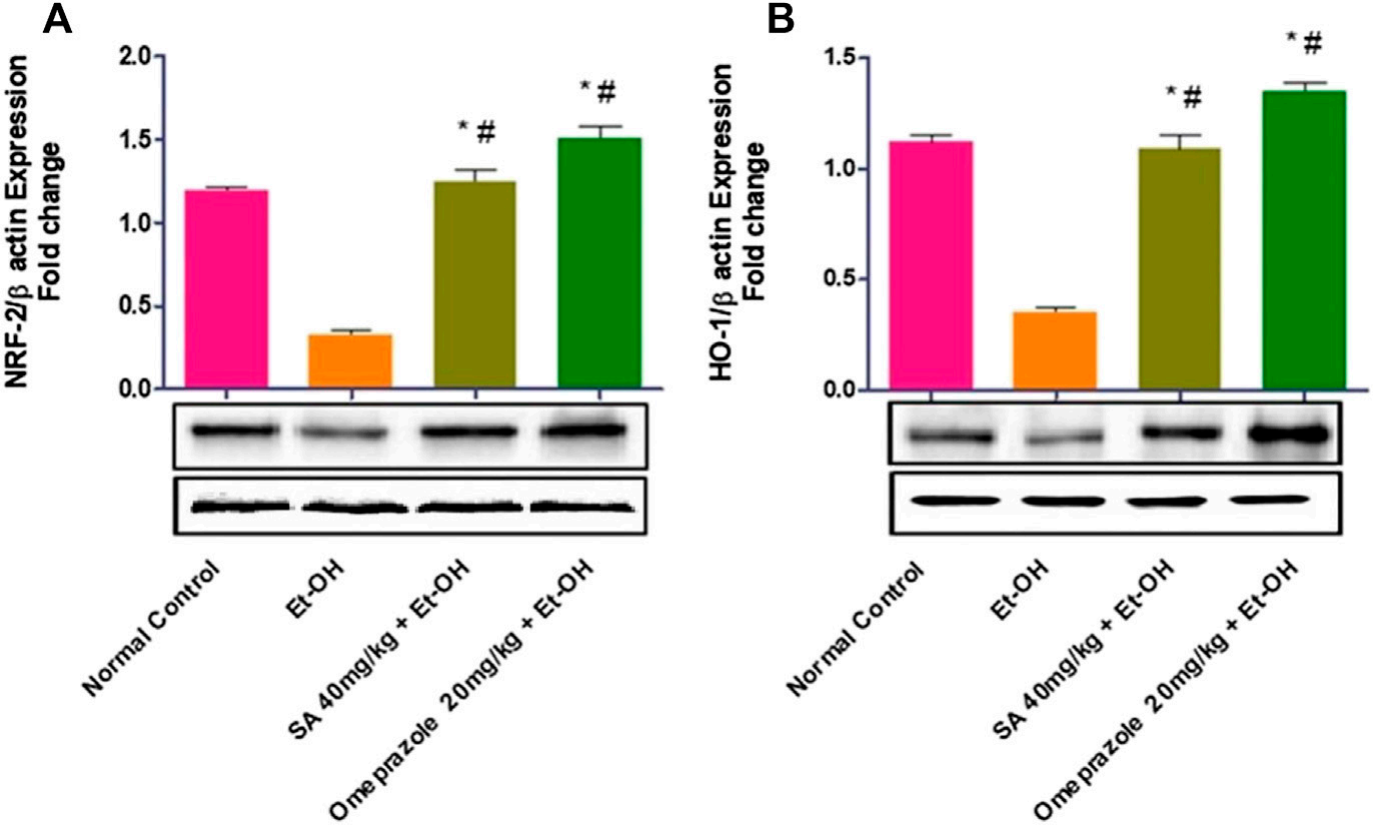
Effect of SA on the protein expression of **(A)** Nrf2 nuclear protein and **(B)** HO-1 cytoplasmic protein in ethanol-treated rats. Data are represented as the mean ± SEM (n = 6) per group. *Denotes significant differences compared to the ethanol-induced gastric ulcer group (*p* < 0.05); #denotes significant differences compared to the normal control.

As illustrated in Figure 6, the expression of the NRF2 (*p* < 0.05) and HO-1 (*p* < 0.05) proteins was significantly reduced in rats with ethanol-induced GUs compared with normal control rats. Chronic administration of SA (40 mg/kg b. w.) and omeprazole (20 mg/kg) led to the upregulation of NRF2 (*p* < 0.05) and HO-1 (*p* < 0.05) in gastric tissues, indicating restoration of the antioxidant defense system.

### Effect of SA Pre-treatment on Histopathological Examination of the Gastric Mucosa in Ethanol-Treated Rats

#### Hematoxylin and Eosin Staining

**FIGURE 7:**
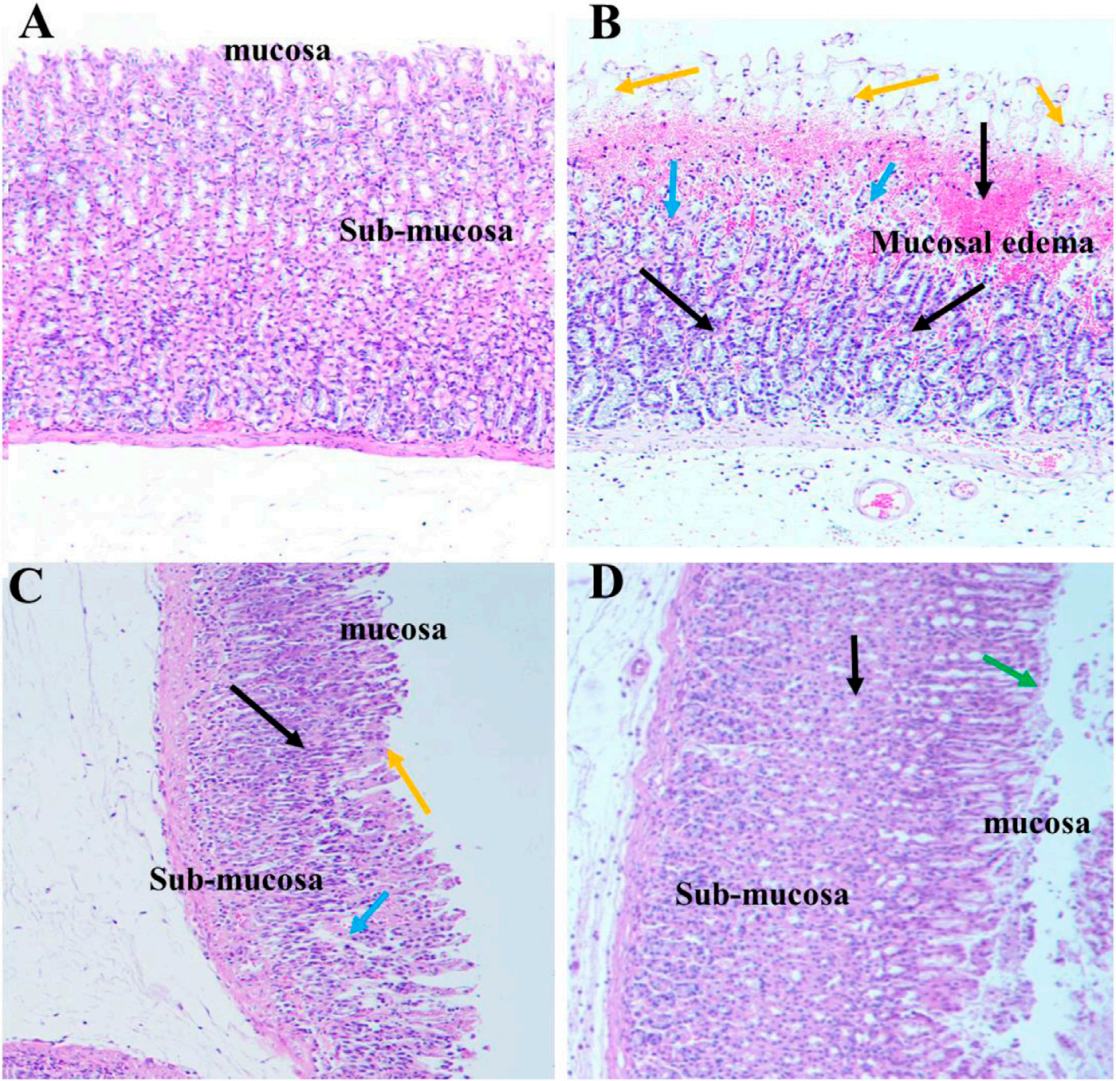
Effect of SA on the gastric epithelium in ethanol-induced gastric mucosal damaged rats (n = 6) **(A)** Normal control rats **(B)** Ulcer control stomach presenting severe mucosal damage (orange arrow) together with deep necrosis (blue arrow), edema, and inflammation of the submucosal layer (black arrow) **(C)** SA exerted a gastroprotective effect, as indicated by reduced mucosal aberration, reduced necrosis of the gastric epithelium, and reduced inflammatory cell infiltration **(D)** Omeprazole treatment exerted a potent gastroprotective effect, as indicated by reduced mucosal aberration, reduced necrosis of the gastric epithelium, reduced inflammatory cell infiltration, and restoration of the mucosal barrier (green arrow).

Histopathological examination of the gastric tissue slides of the ethanol-induced group demonstrated severe depletion of the gastric mucosa with extensive gastric lesions supplemented with intense deterioration, necrosis, and hemorrhages. Moreover, in addition to severe inflammatory cell infiltration, submucosal edema, and depression of gastric pits were also observed in the gastric wall. Conversely, control gastric tissue slides showed normal architecture of the gastric mucosal wall with no signs of deterioration. In turn, SA and omeprazole pre-treatment significantly ameliorated the structural alteration by protecting against the depletion of the gastric mucosa. The reduction in cell infiltration as a result of the ameliorative effect of omeprazole was considerably greater than that observed for SA (Figure 7).

#### Periodic Acid Schiff Staining of Mucosal Glycoproteins

**FIGURE 8:**
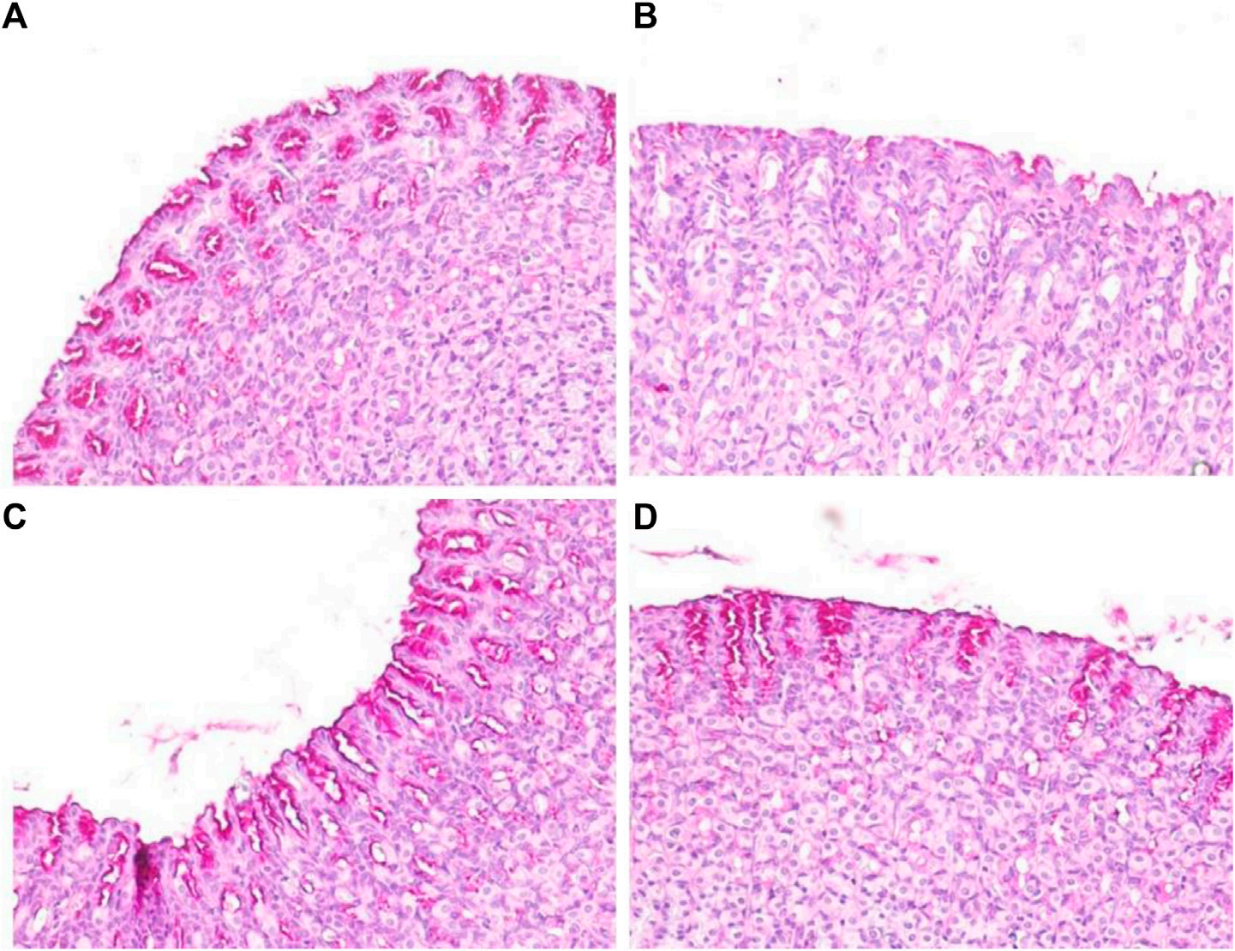
Effects of SA on the PAS staining of secreted gastric glycoproteins in rats with ethanol-induced gastric ulceration (n = 6) **(A)** Normal control exhibiting a normal magenta coloring (black arrow) of the gastric mucus glands **(B)** Ethanol-induced rat with no PAS staining in the mucosa of the ulcer control group, denoting severe mucosal damage **(C)** SA-treated rats exhibited intense PAS staining **(D)** Omeprazole pre-treatment led to intense uptake of the PAS stain, indicating restoration of the mucosal barrier.

Depletion of the gastric mucosal wall was examined by PAS staining. The results clearly demonstrated reduced or no magenta color in the ethanol-induced ulcerated slides, indicating depletion of the mucosal wall barrier. However, normal control slides displayed intense magenta staining, indicating restoration of the mucosal wall barrier. SA and omeprazole significantly restored the intense magenta color, indicating restoration of the gastric mucosal lining, although this was more intense for omeprazole than it was for SA (Figure 8). The quantification of PAS-stained areas in μm^2^/field was performed using the ImageJ software, NIH (Table 3).

**TABLE 3:** PAS-stained areas in the gastric mucosa of rats treated with sinapic acid against ethanol-induced gastric damage, as analyzed using the ImageJ software, NIH.

Groups	Stained area μm^2^/Field ± SEM	Mean % area ± SEM
Normal	2.96 × 10^3^ ± 0.13	8.42 ± 0.37
Ethanol	0.80 × 10^3^ ± 0.03	2.29 ± 0.09
SA 40 mg/kg + Et-OH	3.08 × 10^3^ ± 0.12	8.76 ± 0.34
Omeprazole 20 mg/kg + Et-OH	4.53 × 10^3^ ± 0.11	12.91 ± 0.33

## Discussion

Polyphenols possess several biological activities, including antioxidant and gastroprotective activities (Mota et al., 2009; Sumbul et al., 2011). SA (3,5-dimethoxy-4-hydroxycinnamic acid) is an orally bioavailable phytoconstituent that is present in cereals, fruits, oil, and vegetables and has potent antioxidant, antidiabetic, anticancer, anti-inflammatory, chemopreventive, and antiapoptotic activities (Chen, 2016; Raish et al., 2018a; Bin Jardan et al., 2020). No *in vivo* studies have examined the gastroprotective effect of SA, or the mechanism underlying the gastric protection afforded by it in the GUs of ethanol-induced rats.

In this research, an 80% ethanol (5 ml/kg)-induced gastric ulcer model was established. The ethanol-induced gastric ulcer experimental model represents many aspects of the disorder of human gastric ulceration and is, thus, useful in determining the anti-ulcer capacity of drugs, as well as the likely pathways involved in this process (Kim et al., 2005; Huang et al., 2013; Vijayakumar et al., 2016; Sistani Karampour et al., 2019). In addition, ethanol is one of the most frequent causes of gastric ulceration. Ethanol causes gastric injuries via several pathways, including dehydration, which disrupts mucosal cell barriers, and cytotoxicity. This cytotoxicity contributes to the recruitment of ROS-releasing leukocytes and inflammatory cytokines, all of which may contribute to apoptosis in cells. Strangely, NF-kB plays a key role in the relationship between these disparaging events (Kim et al., 2005).

SA is a nutraceutical that has exhibited cytoprotective, antioxidant, and anti-inflammatory activity in several studies in various *in vivo* models over 1 week or 10 days; therefore, a span of 7 days was selected here (Raish et al., 2019; Bin Jardan et al., 2020). A pilot study was conducted to assess the most appropriate dose of SA, and the data showed that 40 mg/kg had the maximum gastroprotective effect. Three clinical doses were selected based on previous literature, and the dose response was examined for anti-gastric ulcer activity in rats with ethanol-induced gastritis that were orally treated with 10, 20, and 40 mg/kg SA for 7 days. We found that the dose response in terms of GUI and PGE2 levels for the three dose levels of SA, 40 mg/kg had the maximum gastroprotective effect. This was further corroborated by our previous research, which indicated that 40 mg/kg of SA has maximum antioxidant, anti-inflammatory, and antiapoptotic activity (Raish et al., 2018a; Raish et al., 2019; Bin Jardan et al., 2020). Several reports have implicated alcohol in gastric mucosal damage and upper gastrointestinal bleeding (Teyssen and Singer, 2003; Simoes et al., 2019). The anti-ulcer and gastroprotective effects of drugs, natural phytochemicals, and herbs have been commonly assessed using an ethanol-induced gastric lesion rodent model (Simoes et al., 2019). Alcohol intubation causes depletion of the gastric wall mucosal barrier via ROS and cytokine upregulation, which lead to oxidative-stress-induced damage (Abdel-Salam et al., 2001; Liu et al., 2020). In the present investigation, ethanol intubation caused severe oxidative-stress-induced damage in rats with ethanol-induced ulcers; this was consistent with previous literature. Moreover, it was demonstrated that intubation of 80% alcohol reliably induces oxidative stress gastric tissue ulceration, which allows the examination of the gastroprotective effect (Abdelwahab 2013). Furthermore, gross microscopical examination of the stomach demonstrated the presence of hemorrhagic lesions in ethanol-induced rats. Pre-treatment with SA and omeprazole significantly ameliorated ethanol-induced gastric ulceration via anti-inflammatory, anti-oxidative, and antiapoptotic mechanisms, possibly as a result of increased PGE_2_ and NO release. The increase in the GUI in ethanol-induced rats highlights the potent ulcerogenic activity of ethanol, which is consistent with previous literature (Ferraz et al., 1997; Al Batran et al., 2013). The significant reduction in GUI and the enhanced PI of SA suggest a potent antiulcerogenic ability that is comparable to that of omeprazole, a well-established anti-ulcer compound. In addition, SA and omeprazole pre-treatment yielded a reduction in gastric lesions in the gastric wall mucosa; an increase in the gastric pH and mucosa level; and a decrease in the gastric volume, free acidity, and total acidity in ethanol-induced rats. These results agree with those of previous reports (Laloo et al., 2013; Fu et al., 2018; Aziz et al., 2019; Rahman et al., 2020).

This was the first demonstration of the anti-secretagogue, anti-ulcer, and gastroprotective activity of SA in ethanol-induced ulcerated rats. Furthermore, we examined the role of prostaglandins, NO, inflammatory cytokines, inflammatory markers, lipid peroxidation, antioxidant enzymes, apoptotic proteins, and the NRF2/HO-1 pathway to examine the mechanism underlying the anti-ulcer activity of SA. PGE2 and NO are key regulators that maintain the integrity of the gastric mucosal wall defense barrier and healing of GUs (Sanchez-Mendoza et al., 2019). The protective effect of NO is associated with stimulation of the gastric mucosa and bicarbonate secretion, to maintain the blood flow of gastric capillaries, was well as inhibition of inflammation (Tarnawski, 2005; Sanchez-Mendoza et al., 2019). The reduction in PGE2 and NO in the gastric mucosa is one of the primary causes of gastric ulceration (Sanchez et al., 2002). In this investigation, we demonstrated a reduction in the level of PGE2 and NO in the gastric mucosa in ethanol-induced ulcerated rats. However, pre-treatment with SA and omeprazole significantly increased the level of NO and PGE2 in ethanol-induced ulcerated rats (Tsukada et al., 1987; Sanchez et al., 2002; Abdelwahab, 2013). The enhanced secretion of gastric mucus noted in SA- and omeprazole-pre-treated rats could be attributed to the increased production of PGE_2_ and NO in the gastric mucosa. The findings of the present study are consistent with those of many previous reports, which have provided evidence that SA has anti-secretagogue, anti-ulcer, and gastroprotective activity in ethanol-induced GUs in rats (Sistani Karampour et al., 2019; Zhou et al., 2020). The inflammatory response is a key process in the gastric mucosa defense mechanism (Martin and Wallace, 2006). Ethanol encourages inflammation, which triggers the recruitment of macrophages and the accumulation of inflammatory cytokines, such as TNF-α, IL-6, and IL-1β, thus promoting the accumulation of neutrophils at sites of inflammation, the destruction of mucosal barriers, and ROS production (Abdelwahab, 2013; Philpott et al., 2014; Almasaudi et al., 2016; Badr et al., 2019). Ethanol intubation led to an enhancement of TNF-α and IL-6 levels and increased the level of the neutrophil infiltration index marker MPO compared with normal control rats. SA and omeprazole pre-treatment significantly inhibited the elevation of TNF-α, IL-6, and MPO levels. These findings agree with those of earlier reports (Abdelwahab, 2013; Laloo et al., 2013; Sistani Karampour et al., 2019), which shows its potent anti-inflammatory activity. Redox imbalance between ROS generation and antioxidant defense scavenging can cause oxidative stress, with the main source of ROS being infiltrating inflammatory cells (Leitao, Ribeiro et al., 2007). The superoxide radical anions (O_2_−) are produced by neutrophils that react with lipids to produce lipid peroxidation (Kwiecien et al., 2002). The present study demonstrated the impairment of the redox balance that is increased during lipid peroxidation (MDA), as well as depletion of antioxidant enzymes, such as GSH and CAT, in ulcerated gastric tissues. The increased MDA level was attributable to an increase in superoxide radical anions (O_2_) and ROS, and depletion of antioxidant enzymes caused by scavenging by the superoxide radical anion (O_2_−) and ROS (Ito et al., 1998). SA and omeprazole pre-treatment significantly inhibited lipid peroxidation (MDA) and replenished the depleted GSH and catalase activity in ulcerated gastric mucosa. SA has potent anti-lipid peroxidation and free radical scavenging activities (Chen, 2016). These results are consistent with those of previous reports (Chen, 2016; Raish et al., 2018a; Raish et al., 2018b; Raish et al., 2019; Bin Jardan et al., 2020), in that gastric mucosal apoptotic injuries have been shown to by prompted by oxidative stress and inflammatory cytokines via the intrinsic mitochondrial pathway. The oxidative-stress-induced apoptosis controlled by the antiapoptotic Bcl2 and pro-apoptotic Bax and caspase-3 proteins plays a significant role in the disruption of gastric mucosal wall integrity after ethanol intubation (Durkin et al., 2006; Liu et al., 2018; He et al., 2019). Inhibition of apoptosis via downregulation of apoptotic proteins (Bax and caspase-3) and upregulation of Bcl2 expression mitigates gastric lesion recovery (Abdelwahab, 2013; Antonisamy et al., 2015; Aziz et al., 2019; He et al., 2019). SA and omeprazole administration substantially downregulated Bax and caspase-3 and upregulated Bcl2 relative to the ulcerated group. The inflammatory response and oxidative stress are controlled by the NF-kB Rel subfamily transcription regulator, responsible for dimerization, DNA binding, and interaction with inhibitory proteins (Oliveira-Marques et al., 2009). This study revealed that p65 NF-kB expression was increased and IκBα expression was decreased in the gastric mucosa of ethanol-induced ulcerated rats. Conversely, SA and omeprazole pre-treatment downregulated NF-kB and upregulated IκBα. The reduction in the level of NF-κB in SA and omeprazole pre-treated rats may be due to the ROS-scavenging activity of SA and omeprazole, as ROS activate NF-κB via the phosphorylation of IκBα (Lee et al., 2006). This was the first report to demonstrate that SA downregulates NF-kB and inhibits apoptosis in gastric ulcer tissues, thereby ameliorating GUs. Previous reports have demonstrated that antioxidants inhibit the NF-kB activation stimulated by ROS and block the transcription of several inflammatory cytokines (TNFα and IL-6) in ethanol-induced ulcerated rats (Li et al., 2013; Arab et al., 2015).

The Nrf2/HO-1 signaling pathway plays a crucial role in protecting cells from oxidative-stress-induced injuries by restoring endogenous antioxidant enzymes (GSH, CAT, and HO-1) (Yanaka, 2018; Badr et al., 2019). Nrf2 binds to the negative regulator Keap1 and remains inactive in the cytoplasm. Oxidative stress dissociates Nrf2 to from Keap1, and promotes its translocation from the cytoplasm to the nucleus, leading to the activation of Phase II enzymes (Li et al., 2012; Abdelwahab, 2013). HO-1 is most evidently associated with cytoprotection against oxidative stress, as well as protection against apoptosis by curbing ROS (Abdelwahab, 2013; Yanaka, 2018; Rahman et al., 2020). In this study, upregulation of NRF2 and HO-1 was observed in the ethanol-treated group, while the SA- and omeprazole-induced upregulation of NRF2/HO-1 might be responsible for the gastroprotective effect observed in ethanol-induced gastric mucosal injuries in rats. These results are in line with previous findings (Mota et al., 2009; Abdelwahab, 2013; Albaayit et al., 2016; Fu et al., 2018; Yanaka 2018). SA exerted definite antioxidant, anti-inflammatory, and antiapoptotic effects in several experimental pathologies, with the implication of the NF-κB, Nrf2/HO-1, Bcl-2/Bax pathways (Sumbul et al., 2011; Chen, 2016; Raish et al., 2018b; Shahmohamady et al., 2018; Bin Jardan et al., 2020). By mitigating oxidative stress, Nrf2/HO-1 induction can efficiently prevent inflammation. The Nrf2/HO-1 pathway is regarded as the key redox pathway associated with oxidative damage and NF-κB, which is a key regulator of proinflammatory cytokines and markers (Sandireddy et al., 2014; Zhang et al., 2016). NF-κB is upregulated in oxidative stress, and downregulation of NRF2 results in the enhanced production of ROS, thereby upregulating inflammation by augmenting inflammatory cytokines and markers (Pan et al., 2012; Ganesh Yerra et al., 2013).

Our histopathological findings further confirmed that ulcerated tissues display erosion of gastric wall mucous barriers with hemorrhagic lesions, extensive edema, and leukocyte infiltration of the submucosal layer. In contrast, SA and omeprazole pre-treated ulcerated rats exhibited less mucosal damage than did ethanol-induced ulcerated rats without pre-treatment.

## Conclusion

The data of this study revealed that oxidative stress induced by ROS/RNS is a key factor in the pathogenesis of gastric mucosal injury intervened by oxidative stress in an experimental model of ethanol-induced gastritis. The administration of SA at 40 mg/kg reduced oxidative stress biomarkers without modifying the gastric pH. These results revealed an antioxidant effect through Nrf2-mediated HO-1 induction. Furthermore, the gastroprotective effect of SA at a dose of 40 mg/kg may be attributed to the activation of the Nrf2/HO-1 antioxidant pathway and anti-inflammatory pathways via the downregulation of NF-κB, as well as the upregulation of the antiapoptotic protein Bcl-2 and the downregulation of caspase-3 and Bax. All of these mechanisms ultimately maintain normal gastric mucosal barrier integrity. SA exerted a substantial gastroprotective effect against ethanol-induced GUs in rats. This gastro-protection could be associated with the restoration of antioxidant defenses via NRF2/HO-1, downregulation of NF-κB, and inhibition of apoptotic injuries.

## Data Availability

The raw data supporting the conclusions of this article will be made available by the authors, without undue reservation.
